# The Role of Bradykinin Receptors in Hereditary Angioedema Due to C1-Inhibitor Deficiency

**DOI:** 10.3390/ijms231810332

**Published:** 2022-09-07

**Authors:** Wojciech Dyga, Aleksander Obtulowicz, Tomasz Mikolajczyk, Anna Bogdali, Pawel Dubiela, Krystyna Obtulowicz

**Affiliations:** 1 Department of Clinical and Environmental Allergology, Jagiellonian University Medical College, Botaniczna 3, 31-503 Krakow, Poland; 2 Department of Dermatology, Jagiellonian University Medical College, Kopernika 50, 31-501 Krakow, Poland; 3 Department of Internal and Agricultural Medicine, Jagiellonian University Medical College, Skarbowa 1, 31-121 Krakow, Poland; 4 Department of Pathophysiology and Allergy Research, Medical University of Vienna, Waehringer Guertel 18-20, A-1090 Vienna, Austria; 5 Department of Regenerative Medicine and Immune Regulation, Medical University of Bialystok, Waszyngtona 13, 15-269 Bialystok, Poland

**Keywords:** angioedema, bradykinin, bradykinin receptors, C1 inhibitor, HAE

## Abstract

Background: Hereditary angioedema (HAE) is a rare, genetic disease caused by the decreased level or function of the C1 inhibitor. The primary mediator of symptoms in HAE is bradykinin acting through its two receptors, namely receptors 1 (BR1) and 2 (BR2). Although BR2 is well characterized, the role of BR1 remains unclear. Objective: To study the role of bradykinin receptors 1 (BR1) in the etiopathogenesis of HAE. Methods: A total of 70 individuals, 40 patients with HAE, and 30 healthy subjects were recruited to the study. HAE was diagnosed in accordance with the international guideline. The level of bradykinin receptors was determined in populations of CD3^+^, CD4^+^, CD8^+^, and CD14^++^CD16^−^, CD14^++^CD16^+^ monocytes. In addition, the level of disease activity-specific markers was measured. Results: There were statistically significant differences in the subpopulation of lymphocytes and monocytes between patients with HAE compared to healthy subjects. The level of BR1 and BR2 on PBMCs was comparable in healthy subjects and HAE patients during remission with significant overexpression of both receptors, triggered by HAE attack. Moreover, a significant increase in TNF-alpha and IL-1 plasma levels was observed among HAE patients. Conclusions: BR1 expression may play an important role in the pathomechanism of HAE.

## 1. Introduction

Hereditary angioedema (HAE) is a rare disorder caused by either a lack or dysfunctional C1-inhibitor protein with estimated prevalence of 1 in 50,000 [1,2]. C1-inhibitor deficiency is caused by mutations in the *SERPING1* gene. The symptoms are not mutation specific and vary in severity, location, or duration. The most frequent location is the skin, but the organs involved include also upper airways, genitals, or gastrointestinal tract. Attacks last 2 to 5 days, usually progressing and resolving if not treated with on-demand therapy [3,4].

C1-inhibitor deficiency causes a broad spectrum of homeostasis dysregulation in a plasma bradykinin-forming cascade triggering overproduction of bradykinin (BK) due to uninhibited effects of activated factor XII (FXII) and plasma kallikrein [5]. BK is a direct culprit of HAE symptoms leading to increased vascular permeability and thus angioedema [6,7]. To play such a role, BK activates two types of receptors, namely bradykinin receptor 1 (BR1) and bradykinin receptor 2 (BR2) [8,9]. In the human genome, bradykinin receptors 1 and 2 are located next to each other in the 14q32 region [10]. Although genetically very close, they represent completely different molecular capabilities and functions. Bradykinin receptor 1 is believed to be irrelevant in HAE because of its subnanomolar affinity to Lys-des-Arg^9^-BK, a kinin metabolite [10]. It was also proved to be activated upon certain conditions. Interestingly, the injection of bacterial lipopolysaccharide in laboratory animals can stimulate BR1 which leads to responses such as hypotension, vasodilation, and increased vascular permeability observed also during HAE attacks [11,12]. In opposite, BR2 presenting as the constitutive receptor with a high affinity for BK was found as the major player in symptomatology, and thus, a target for HAE treatment [3].

The critical reactions of the contact pathway in HAE leading to bradykinin-mediated angioedema occur locally at the surface of endothelial cells [13]. In vitro studies highlight the endothelial cell membrane as the location for bradykinin production. First, FXII interacts with the complex of the urokinase plasminogen activator receptor and cytokeratin 1 present on endothelial cell surfaces [14]. Subsequently, high molecular weight kininogen–kallikrein complexes bind to the receptor of C1q and cytokeratin 1 on the endothelial cell surface [15]. All of those elements are placed in the center of bradykinin production. It is well known that interaction between BK and BR2 results in increased vascular permeability, thus mediating swelling. The role of BR1 in the process remains unclear. Interestingly, there are some studies on other diseases aiming to explain the interplay between bradykinin and its receptors; i.e., the study from Marketou et al. The authors clearly showed that not only BR2 but also BR1 plays an important role in hypertension. The study performed on monocytes from patients with essential hypertension compared with healthy individuals revealed that both receptors are elevated in essential hypertension and contribute to the development of target organ damage [16].

Thus, the study has been performed on a well-characterized cohort of HAE patients to figure out whether BR1 plays any role in the pathomechanism of the disease. We have also studied the expression of BR2 on a selected cell subpopulation during an HAE attack and remission. In addition, we aim to evaluate some disease markers to figure out their presence depending on disease activity.

## 2. Results

### 2.1. Comparison of Lymphocytes and Monocytes Subpopulations Distribution in HAE Patients during the Attack and Remission

**Figure 1:**
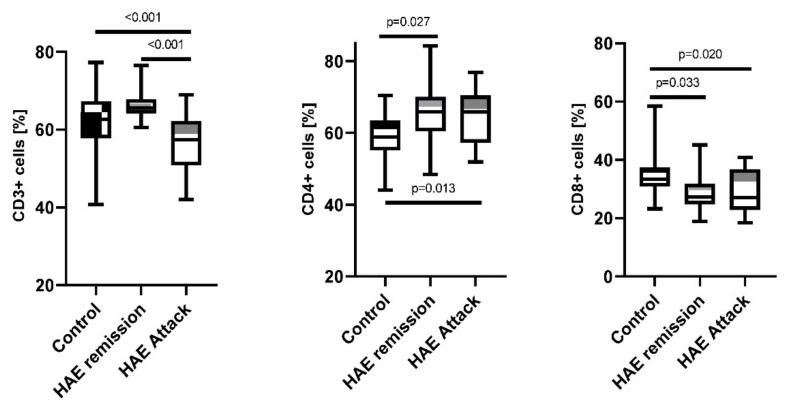
Lymphocytes subpopulations distribution among HAE patients during attack and in remission compared to the healthy control subjects.

Comparison of lymphocytes subpopulations distribution between examined groups showed significantly increased subpopulation of CD4^+^ in HAE patients during the attack (65.8%; 51.9–76.8%, *p* = 0.013) and in remission (66.1%; 48.4–84.2%, *p* = 0.027) in comparison to healthy subjects (58.8%; 44.0–70.4%). On the contrary, the number of CD3^+^ was significantly lower in patients during HAE attacks (57.4%; 42.0–68.9%) when compared to remission state (65.6%; 60.5–76.5%, *p* < 0.001) and healthy subjects (62.6%; 40.7–77.3%, *p* < 0.001). The same statistically significant decrease in the number of CD8^+^ cells was observed for samples collected during the attack (27.1%; 18.4–40.8%, *p* = 0.020) and those that were taken in the remission (27.2%; 18.9–45.1%, *p* = 0.033) compared to the healthy subjects (33.3%; 23.2–58.4%, Figure 1/Table 1).

**Figure 2:**
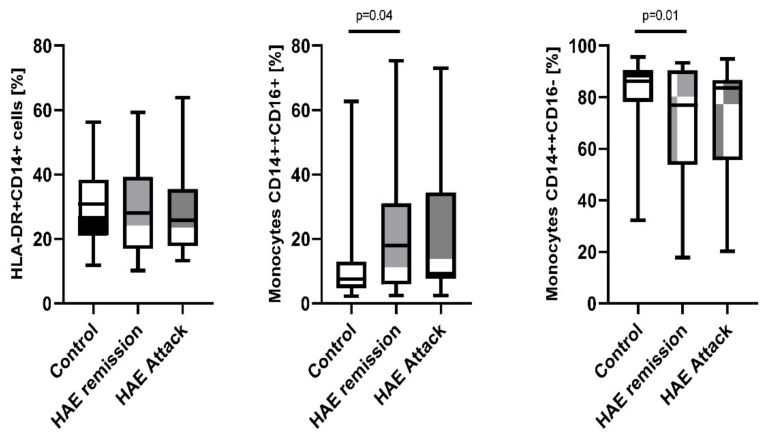
Monocytes subpopulations distribution among HAE patients during attack and in remission compared to the healthy control subjects.

When comparing monocytes there were no significant differences among the groups in the number of monocytes (HLA-DR^+^CD14^+^ cells). However, a significant difference was found between healthy subjects and HAE patients during remission (7.6%; 2.3–62.7% vs. 19.4%; 2.5–75.3%, *p* = 0.04) in the number of CD14^++^CD16^+^. A similar observation was made when comparing CD14^++^CD16^−^ in the group of healthy subjects and HAE patients in remission (86.3%; 32.4–95.6% vs. 75.4%; 17.9–93.4%, *p* = 0.01, Figure 2/Table 1).

### 2.2. Disease Activity Correlation with BR1

**Figure 3:**
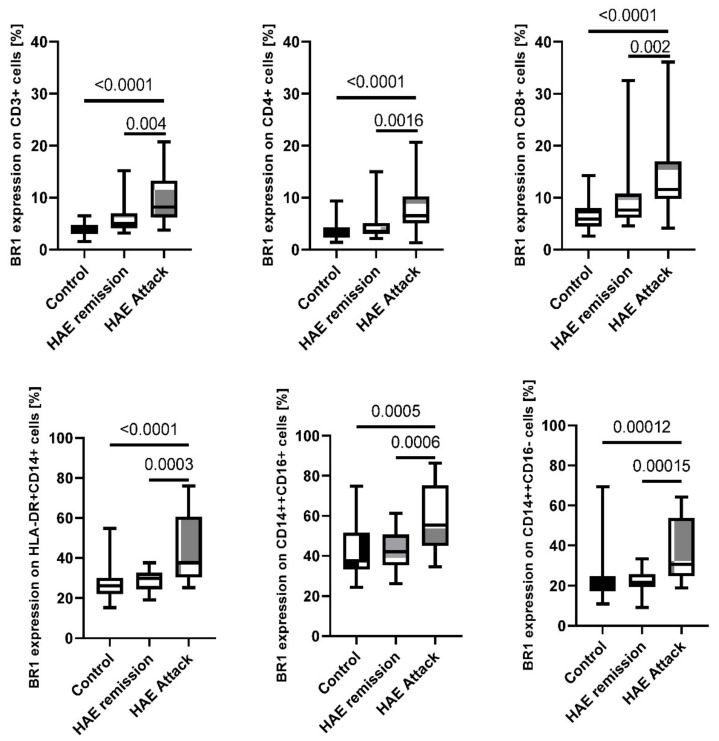
BR1 expression on subpopulations of lymphocytes and monocytes among HAE patients during attack and in the remission compared to the healthy control subjects.

A significant correlation between disease activity (HAE attacks) and BR1 expression was found on CD3^+^, CD4^+^, and CD8^+^ cells. Percentage of all the T cells subtypes expressing BR1 in the patients during attack (CD3^+^ 8.19%; 3.73–20.71%; CD4^+^ 6.50%; 1.33–20.65%; and CD8^+^ 11.56%; 4.15–36.1%) was doubled or tripled in comparison to the remission (CD3^+^ 5.06%; 3.20–15.17%; *p* = 0.004; CD4^+^ 3.55%; 2.16–14.97%; *p* = 0.0016; and CD8^+^ 7.62%; 4.56–32.54%; *p* = 0.002) and healthy subjects (CD3^+^ 3.60%; 1.54–6.48%; *p* < 0.0001; CD4^+^ 2.88%; 1.39–9.36%; *p* < 0.0001; and CD8^+^ 5.90%; 2.58–14.28%; *p* < 0.0001, Figure 3/Table 2).

Moreover, monocytes increased the expression of BR1 during disease manifestation. Patients with HAE during attack had significantly higher expression of BR1 on the HLA-DR^+^CD14^+^ (37.65%; 25.29–76.03%) comparing to the remission state (29.94%; 19.20–37.60%; *p* = 0.0003) and healthy subjects (26.19%; 15.31–54.91%; *p* < 0.0001). It was also noted for CD14^++^CD16^+^ cells during HAE attack (55.39%; 34.52–86.40%) compared to the remission state (42.15%; 26.25–61.40%; *p* = 0.0006) and healthy subjects (37.50%; 24.34–74.77%; *p* = 0.0005). Similar trend was observed for BR1 expression on CD14^++^CD16^−^ that also revealed a significant difference between HAE patients during the attack (30.63%; 18.99–64.21%) and the remission state (21.83%; 9.15–33.43%; *p* = 0.00015) and healthy subjects (19.80%; 10.97–69.38%; *p* = 0.00012; Figure 3/Table 2).

### 2.3. Disease Activity Correlation with BR2

**Figure 4:**
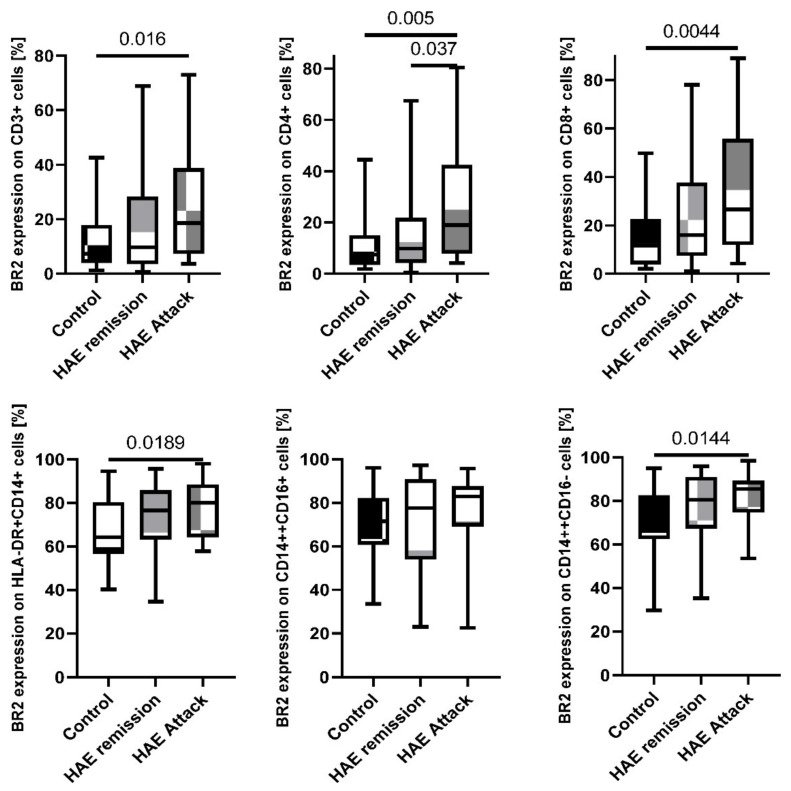
BR2 expression on subpopulations of lymphocytes and monocytes among HAE patients during attack and in the remission compared to the healthy control subjects.

Significant correlation between disease activity (HAE attacks) and bradykinin receptor 2 expression was found on CD3^+^, CD4^+^, and CD8^+^ cells. The percentage of all the T cell subtypes expressing BR2 in the patients during attack (CD3^+^ 18.57%; 3.73–73.05%; CD4^+^ 18.98%; 4.23–80.65%; and CD8^+^ 26.59%; 4.21–88.99%) was multiplied in comparison to the remission (CD3^+^ 9.70%; 0.78–68.89%; *p* = 0.062; CD4^+^ 9.78%; 0.56–67.48%; *p* = 0.037; and CD8^+^ 15.90%; 0.99–78.13%; *p* = 0.07) and healthy subjects (CD3^+^ 7.35%; 1.26–42.60%; *p* = 0.016; CD4^+^ 7.47%; 1.82–44.52%; *p* = 0.005; and CD8^+^ 11.67%; 2.04–49.78%; *p* = 0.0044, Figure 4/Table 2).

Moreover, monocytes increased the expression of BR2 during disease manifestation. During the attack, patients with HAE presented higher expression of BR2 on the HLA-DR^+^CD14^+^ (80.04%; 57.90–97.95%) comparing to remission state (76.58%; 34.65–95.69%; *p* = 0.386) and healthy subjects (64.29%; 40.26–94.50%; *p* = 0.0189). It was also noted for CD14^++^CD16^+^ cells during HAE attack (82.90%; 22.56–95.74%) compared to the remission state (77.61%; 22.97–97.18%; *p* = 0.655) and healthy subjects (71.58%; 33.50–96.05%; *p* = 0.058), with differences that were also not statistically significant. A similar trend was observed for BR2 expression on CD14^++^CD16^−^ for HAE patients during the attack (85.49%; 53.60–98.46%) in comparison to remission state (80.55%; 35.28–95.97%; *p* = 0.457) and healthy subjects (68.96%; 29.76–95.00%; *p* = 0.0144; Figure 4/Table 2).

### 2.4. Disease Specific Markers during the Attacks and the Remission State

**Figure 5:**
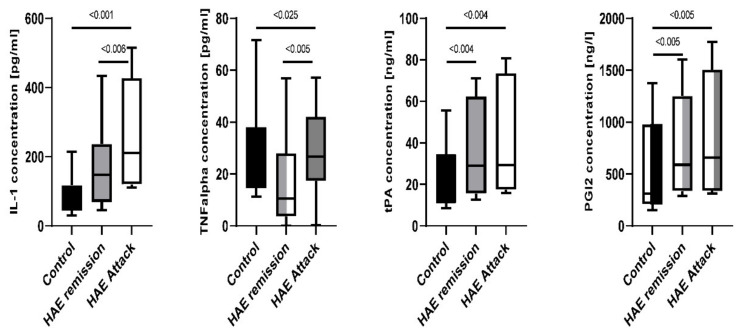
Disease-specific markers expression among HAE patients in attack and during remission compared to the healthy control subjects.

All tested markers, namely: IL-1, TNF-alpha, tPA, and PGI2 were significantly elevated in patients with HAE compared to the healthy subjects. IL-1 level during attacks reached 210.9 pg/mL (110.6–515.2 pg/mL) and was higher comparing to the remission state 148.1 pg/mL (45.3–434.1 pg/mL, *p* < 0.006) and healthy subjects 58.3 pg/mL (30.2–214.5 pg/mL, *p* < 0.001). TNF alpha concentration during attack was 26.8 pg/mL (0.3–57.2 pg/mL), significantly higher than during remission; 10.5 pg/mL (0.1–56.9 pg/mL, *p* < 0.025) and in healthy subjects 17.6 pg/mL (11.2–71.6 pg/mL, *p* < 0.05). tPA was significantly higher in patients with HAE both during the attack 29.3 pg/mL (15.8–80.8 pg/mL) and remission (29.0 pg/mL, 12.6–71.2 pg/mL) in comparison to healthy subjects (12.31 pg/mL, 8.7–55.6 pg/mL, *p* = 0.004 and *p* = 0.04, respectively). Moreover, PGI2 was significantly elevated among HAE patients regardless of disease activity. It was 659.6 pg/mL (312.8–1772.2 pg/mL) during attack and 589.0 pg/mL (288.9–1600.5 pg/mL) in the remission. Subjects without HAE had 309.8 pg/mL (151.5–1377.4 pg/mL, *p* < 0.005 and *p* < 0.005, respectively, Figure 5/Table 3).

## 3. Discussion

While the bradykinin receptor 2 role is well established in HAE and is applied to several treatment approaches, the role of BR1 remains unclear. Following some hints from other branches of medicine i.e., dermatology or cardiology we aimed to figure out whether BR1 can play a role in the etiopathogenesis of the disease. We tested BR1 and BR2 expression on the monocytes and lymphocytes in HAE attack and during remission. We also collected data on the cell characteristics and disease-specific markers to find out whether there is a significant difference between controlled and active angioedema.

The cells distribution revealed in our experiments provides new highlights to a common understanding of HAE. The amount of CD3^+^ cells is significantly lower during the HAE attack despite not being affected by HAE itself. It is opposite to other lymphocyte subtypes, namely CD4^+^ and CD8^+^ that are elevated and decreased by the presence of HAE regardless of activity, respectively. Disruption in the number of lymphocytes when comparing HAE patients and healthy subjects can be somehow surprising. Since the cells mediate adaptive immune responses, they seem to be uninvolved in HAE [17]. Only a few studies have reported abnormal T and B cell counts or abnormal distribution of T cell surface IgG-receptors [18,19].On the contrary, the study from Lopez-Lera et al. on the profiling of the RNA expression of peripheral blood mononuclear cells (PBMC) from C1-INH-HAE patients did not prove alterations in the expression pattern of PBMC in association with frequency and severity of disease [20]. We do believe that our results provide some clues on the role of lymphocytes that differ among this study cohorts. Perhaps, T cells can be involved in the metabolism of plasminogen by glycosylphosphatidylinositol-anchored protein as suggested by Castellano et al. [21]. Our results add to the hypothesis proposed in a perfect review by Ferrara et al., that cells of adaptive immunity could have a role in the regulation of the severity of this disease. Probably, the HAE attack duration can be altered by the cross-play between immune cells producing vasoactive mediators, including bradykinin, histamine, complement components, or vasoactive mediators. Such molecules activate certain immune cell subtypes contributing to vascular endothelial processes that lead to hyperpermeability and tissue edema [17].

The BR1 is an exceptional G protein-coupled receptor that is inducible in vascular cells, notably under the influence of tissue injury, cytokines, and the signaling systems [22]. Thus, we expected that it will be also elevated in patients with HAE during an attack. Indeed, our results clearly demonstrate significant upregulation in all lymphocytes and monocytes subpopulations, respectively. Interestingly, the level of BR1 expression among HAE patients without an attack was similar to healthy subjects. It explains the nature of the receptor, tightly regulated on the transcriptional level under stress conditions. Our observation is supported by previous studies showing that BR1 is synergistically upregulated in human umbilical vein endothelial cells by cotreatment with tumor necrosis factor-α and interferon-γ. It can be also induced by cytokines such as IL-1 [23]. Similar observations were made by Bossi et al. who have performed the only study on the role of BR1 in HAE so far. Both, in vitro endothelial cells transwell model system and in vivo rat’s mesentery microvessels methodology were approached to study vascular leakage. The authors found that a mixture of BR1 and BR2 antagonists prevent totally the permeabilizing effect caused by C1-inhibitor deficiency in patients’ plasma samples collected during attacks [24]. Adding presented results to the evidence generated by other groups, we claim that the role of the BR1 in triggering and/or maintaining HAE symptoms seems to be more important than had been previously believed and further studies on bigger cohorts should be performed, ideally also with antagonists of BR1 given during the HAE attack. Unfortunately, currently, there is no such molecule clinically available. 

BR2 is believed to be widely and constitutively expressed on a number of cells [25]. Our results are contradictory to such an observation suggesting that during HAE attacks, BR2 is also significantly overexpressed on all tested lymphocytes and monocytes subpopulations besides the CD14++CD16+ monocyte subset. Besides our observation, there are no further studies on BR2 expression during an HAE attack. Thus, we can only speculate on this finding with some clinical observations in line with a molecular phenomenon described in the manuscript. Real-world evidence data for BR2 antagonist, icatibant, revealed that it is beneficial for patients to administer the drug as soon as the prodromal symptoms appear [26]. If not, some patients require repetition of the dose administration [27]. This finding can be probably explained by our findings. Once the HAE attack is progressing, cytokines, i.e., IL-1, contribute to BR2 overexpression, as shown in mRNA levels in the study by Koumbadinga et al. [23]. Such dynamics, with transition among bradykinin receptors proposed by Marceau et al., can further explain the heterogeneity of HAE disease among patients with the same mutation in the *SERPING1* gene [28]. The results are also in line with the results obtained by Lee et al. They found that some neurons can upregulate BR2 expression after stimulation with neurotrophic factors or after nerve crush injury [29]. Even more interesting are the results obtained by Zhang et al. that found BR2 upregulation in a murine in vitro model of chronic airway inflammation [30]. A critical factor triggering the receptor expression was TNF-alpha which is not only known to be present during HAE attacks but was also found to be increased in our cohort. Interestingly, TNF-alpha is a well-known inflammatory cytokine produced by macrophages/monocytes during acute inflammation that can orchestrate CD4+ lymphocytes to produce, i.e., IFNγ [31]. Both molecules stimulate BR1 expression which again shows how accruing the molecules cascade can be once triggered.

Our results on disease-specific markers are in line with previous findings [32]. IL-1, TNF alpha, t-PA, and PGI2 were found to be elevated in HAE patients during an attack [33,34]. Notably, IL-1 and TNF alpha were also significantly elevated in remission in comparison to healthy subjects which is in line with previous findings from Arcoleo et al. [35]. This fact should be further studied, especially to understand whether elevation of these inflammation mediators is involved in upcoming of the attack. They have been reported to stimulate endothelial cells and augment activation of the prekallikrein–high molecular weight kininogen complex, suggesting a possible role in triggering HAE attacks. Our data are not in power to explain the fact [36].

In conclusion, the results of the research indicate that dynamics in bradykinin receptors expression can be one of the missing players in understanding the HAE mechanism. The study provides new insight into the biological role of BR1 in HAE pathomechanism. It also suggests that BR2 expression can be induced by special environmental conditions that are present during an HAE attack. Perhaps, our cellular approach can explain previously obtained phase III clinical study results with icatibant and plasma-derived C1-INH. Although approved drugs for HAE on-demand treatment caused symptom relief within 15 to 180 min after treatment, complete resolution was achieved only in several hours [37,38]. We believe that our data can contribute to better patient management, where bradykinin receptors expression can be taken as the factor for antagonist dose administration or stimulate new clinical trials targeting not only BR2 but also BR1. However, studies on bigger and well-described cohorts and/or perfect clinical trials need to be performed to prove our hypothesis and results obtained on the cellular level.

## 4. Materials and Methods

### 4.1. Chemicals and Reagents

All reagents were purchased from Sigma-Aldrich (Saint Louis, MO, USA) unless stated otherwise.

### 4.2. Study Participants and Blood Collection

All patients who entered the study were diagnosed with HAE in accordance with international WAO/EAACI guidelines [39]. Forty HAE patients (male = 14, female = 26; age 21–70, median 34) and 30 healthy controls aged (18–61, median 36) and sex rate (male = 10, female = 20) were recruited to the study to eliminate any significant bias. Healthy subjects were included proving negative family history of HAE and normal serum levels of C1-INH, functional activity of C1-INH, and serum C4 levels.

Blood samples were collected from all healthy subjects and the HAE patients in remission meaning without attack for a minimum of 14 days. In addition, in 20 patients during HAE attack, blood samples were taken. Performed tests included aC1-INH, fC1-INH, and serum C4 levels and were performed in a certified laboratory. Experiments during attack included in addition evaluation of selected HAE markers in plasma (IL-1, TNF alpha, tPA, PGI2) with a standardized ELISA method (Sunred Biological Technology, Shanghai, China).

To estimate the severity and the burden of the disease we followed a consensus report obtained by panel experts. Each patient was interviewed according to the questionnaire presented in Table 2 of the guideline [40]. Our cohort was stratified from mild to moderate or severe group by the evaluation performed by one experienced allergist managing HAE patients for >20 years. The study was approved by the local Ethics Committee of Jagiellonian University in Cracow (104/B2014, 22 May 2014) and conducted in accordance with the Declaration of Helsinki. Patients gave their written informed consent. All experiments were performed in accordance with the relevant guidelines and regulations. More detailed information about patients’ clinical characteristics is shown in Table 4.

### 4.3. Cell Staining and Flow Cytometry

Peripheral Blood Mononuclear cells (PBMCs) were isolated from the peripheral blood of the donors by density gradient separation with Ficoll-Hypaque (PAN BiotechGmbh, Aidenbach, Germany). Cell staining was performed as previously described [41]. Briefly, to assess the expression of the BR1, cells were stained for 30 min at 4 °C with anti-BDKRB1 PE-Cy7 (Polyclonal, Bioss Antibodies, Woburn, MA, USA). To assess the expression of the BR2 cells were first permeabilized for 30 min at 4 °C using the Fixation/Permeabilization set (eBioscience, San Diego, CA, USA) and stained for 30 min at 4 °C with rabbit monoclonal antibodies anti-BDKRB2 (Clone: EPR5646, Abcam, Cambridge, UK) combined with mouse monoclonal secondary antibody anti-rabbit IgG PE (Clone 2A9, Abcam).

Cells were analyzed using BD FACSVerse^TM^ flow cytometer with BD FACSuiteTM software (BD Biosciences, San Jose, CA, USA) and FlowJo software (FlowJo, Ashland, OR, USA). Cell viability was determined by using the Fixable Viability Dye eFluor 780 (eBioscience, San Diego, CA, USA). A standardized gating strategy was employed, as previously described by Obtulowicz et al. [41].

### 4.4. Statistical Analyses

All the analyses were performed with Statistics v13.0 (Tibco Software Inc., Tulsa, OK, USA). The data were presented as the median and range (min; max). Box-and-whisker plots showed the median, interquartile range (IQR), and minimum and maximum values. Normality of the data was assessed using the Shapiro–Wilk test. The Mann–Whitney test was used to compare patients and control. Spearman’s test was used to measure the strength of association between two variables. *p*-values of <0.05 were considered statistically significant.

## Figures and Tables

**Table 1:** Cells characteristics in the tested cohorts. Data are presented as median and range (Q1; Q3). Statistical significance of the results was analyzed with a Mann–Whitney test.

Subpopulation of Cells	Healthy Control Group Median (%)	HAE Patients during Remission (% of Cells)	HAE Patients during Attack (% of Cells)	*p*-Value Remission vs. Control	*p*-Value Attack vs. Control
**CD3^+^**	62.6 (59.2–66.8)	65.95 (64.0–68.7)	57.35 (52.1–61.7)	Non sign	*p* < 0.001
**CD4^+^**	58.8 (56.0–63.0)	66.1 (59.3–71.9)	65.8 (57.5–70.3)	*p* = 0.027	*p* = 0.013
**CD8^+^**	33.3 (30.8–37.3)	27.0 (23.9–34.2)	27.4 (23.1–36.8)	*p* = 0.033	*p* = 0.020
**Monocytes** HLA-DR^+^CD14^+^	30.9 (21.8–38.1)	28.1 (19.3- 36.6)	25.85 (18.3–34.1)	Non sign	Non sign
**Monocytes intermediate subset** CD14^++^CD16^+^	7.65 (4.8–12.7)	19.4 (7.2–36.5)	9.3 (7.7–23.5)	*p* = 0.04	Non sign
**Monocytes classic subset** CD14^++^CD16^−^	86.3 (79.0–90.3)	75.4 (53.2–88.65)	83.7 (69.2–86.4)	*p* = 0.01	Non sign

**Table 2:** Median of bradykinin receptors 1 and 2 expression on lymphocytes and monocytes. Statistical significance of the results was analyzed with a Mann–Whitney test.

	Healthy Control Group (% of Cells)	HAE Patients during Remission (% of Cells)	HAE Patients during Attack (% of Cells)	*p*-Value Attack vs. Control
**BR1 level on CD3^+^** (min-max)	3.6 (3.10–4.65)	5.06 (4.09–5.97)	8.19 (6.41–11.33)	<0.0001
**BR1 level on CD4^+^** (min-max)	2.88 (2.30–4.25)	3.55 (2.89–4.13)	6.50 (5.14–9.35)	<0.0001
**BR1 level on CD8^+^** (min-max)	5.90 (4.50–7.90)	7.62 (6.05–9.83)	11.56 (9.83–15.90)	<0.0001
**BR2 level on CD3^+^** (min-max)	7.35 (4.22–17.33)	9.70 (3.65–29.79)	18.57 (7.66–36.58)	0.016
**BR2 level on CD4^+^** (min-max)	7.47 (3.98–14.75)	9.78 (4.59–23.59)	18.98 (8.60–35.52)	0.005
**BR2 level on CD8^+^** (min-max)	11.67 (4.27–19.83)	15.90 (8.32–39.13)	26.59 (12.49–54.13)	0.0044
**BR1 level on HLA-DR^+^CD14^+^** (min-max)	26.19 (22.12–29.51)	29.94 (24.73–32.54)	37.65 (30.57–59.72)	<0.0001
**BR1 level on CD14^++^CD16^+^** (min-max)	37.50 (33.46–48.89)	42.15 (35.45–50.58)	55.39 (45.14–74.41)	0.0005
**BR1 level on CD14^++^CD16^−^** (min-max)	19.80 (17.58–24.32)	21.83 (19.84–25.77)	30.63 (25.72–51.98)	0.00012
**BR2 level on HLA-DR^+^CD14^+^** (min-max)	64.29 (57.56–79.66)	76.58 (65.26–85.17)	80.04 (67.91–87.94)	0.0189
**BR2 level on CD14^++^CD16^+^** (min-max)	71.58 (61.56–82.08)	77.61 (55.44–90.31)	82.90 (72.75–86.99)	Non sign
**BR2 level on CD14^++^CD16^−^** (min–max)	68.96 (63.52–82.51)	80.55 (67.51–90.71)	85.49 (76.47–89.06)	0.0144

**Table 3:** Correlation between disease-specific markers and HAE activity.

	Healthy Control Group	HAE Patients during Remission	HAE Patients during Attack	*p*-Value (Remission vs. Attack)	*p*-Value (Attack vs. Control)
**IL-1** **[pg/mL]** (min-max)	58.29 (45.4–115.4)	**148.1** **(72.1–231.5)**	210.9 (120.1–420.9)	<0.006	<0.001
**TNF alpha** **[pg/mL]** (min-max)	17.6 (14.6–36.3)	10.53 (4.0–26.91)	26.79 (17.9–34.7)	<0.005	<0.025
**t-PA** **[ng/mL]** (min-max)	12.31 (11.0–29.6)	**29.0** **(16.1–60.7)**	29.3 (17.5–71.3)	0.45	<0.004
**PGI2** **[ng/L]** (min-max)	309.8 (217.5–823.1)	**589.0** **(339.3–1213.7)**	659.6 (346.2–1499.1)	0.28	<0.005

**Table 4:** HAE Patients’ characteristics.

Number of HAE patients	40
Sex male/female *n* (%)	14 (35%)/26 (65%)
Median age (range)	34 (21–70)
**Disease characteristics**	
Family history *n* (%)	30 (75%)
HAE Type *n* (%) Type 1 Type 2	35 (88%) 5 (12%)
HAE symptoms Mild to Moderate, *n* (%) Severe, *n* (%)	21 (52%) 19 (48%)
**Biomarker levels**	
aC1-INH median (g/L) Type 1 Type 2	0.12 0.4
fC1-INH (%) median	20.6
C4 (g/L) median	0.05

aC1-INH—antigen C1-INH concentration (g/L); reference range: 0.21–0.36 (g/L), fC1-INH—functional activity of C1-INH (%); reference range: 70–130%, C4—antigen C4 concentration (g/L); reference range 0.1–0.38 (g/L).

## Data Availability

The data presented in this study are available on request from the corresponding author. The data are not publicly available due to restrictions, e.g., privacy.
